# Associations between Oral Glucose-Lowering Agents and Increased Risk for Life-Threatening Arrhythmias in Patients with Type 2 Diabetes Mellitus—A Literature Review

**DOI:** 10.3390/medicina59101760

**Published:** 2023-10-02

**Authors:** Cristina Tudoran, Mariana Tudoran, Catalina Giurgi-Oncu, Ahmed Abu-Awwad, Simona-Alina Abu-Awwad, Florica Voiţă-Mekereş

**Affiliations:** 1 Department VII, Internal Medicine II, Discipline of Cardiology, University of Medicine and Pharmacy “Victor Babes” Timisoara, E. Murgu Square, Nr. 2, 300041 Timisoara, Romania; tudoran.cristina@umft.ro; 2 Center of Molecular Research in Nephrology and Vascular Disease, Faculty of the University of Medicine and Pharmacy “Victor Babes” Timisoara, E. Murgu Square, Nr. 2, 300041 Timisoara, Romania; 3 County Emergency Hospital “Pius Brinzeu”, L. Rebreanu, Nr. 156, 300723 Timisoara, Romania; catalina.giurgi@umft.ro (C.G.-O.); ahm.abuawwad@umft.ro (A.A.-A.); alina.abuawwad@umft.ro (S.-A.A.-A.); 4 Discipline of Psychiatry, Department of Neuroscience, University of Medicine and Pharmacy “Victor Babes” Timisoara, Eftimie Murgu Place Nr. 2, 300041 Timisoara, Romania; 5 Department XV, Discipline of Orthopedics—Traumatology, “Victor Babes” University of Medicine and Pharmacy, Eftimie Murgu Square, No. 2, 300041 Timisoara, Romania; 6 Research Center University Professor Doctor Teodor Șora, “Victor Babes” University of Medicine and Pharmacy, Eftimie Murgu Square, No. 2, 300041 Timisoara, Romania; 7 Doctoral School, “Victor Babes” University of Medicine and Pharmacy, Eftimie Murgu Square, No. 2, 300041 Timisoara, Romania; 8 Department of Morphological Disciplines, Faculty of Medicine and Pharmacy, University of Oradea, 1 Universitatii Street, 410087 Oradea, Romania; mekeres_florina@yahoo.com

**Keywords:** type 2 diabetes mellitus, ventricular arrhythmias, sudden cardiac arrest, glucose lowering drugs

## Abstract

*Background and Objectives*: The relationship between type 2 diabetes mellitus (T2DM) and cardiovascular (CV) morbidity and mortality is well-established. Ventricular arrhythmias (VA) are frequently diagnosed in patients with T2DM, especially in those with associated coronary syndrome, non-ischemic dilated cardiomyopathy (NIDCM), and heart failure (HF). In these patients, VA and sudden cardiac arrest (SCA) are considered responsible for more than 50% of CV deaths. Newly developed glucose-lowering agents (GLA) seem not only to ameliorate CV morbidity and mortality, but also to reduce the risk of VA and SCA. *Materials and Methods*: We researched the medical literature on Pub-Med, Clarivate, and Google Scholar for original articles published in the last five years that debated the possible effects of various GLA on ventricular arrhythmias. Results: We identified nineteen original articles, nine of them debating the antiarrhythmic effects of sodium-glucose cotransporter-2 inhibitors (SGLT2i); *Conclusions*: The results concerning the impact of various GLA on VA/SCA were heterogeneous depending on the pharmacological class studied, with some of them having neutral, positive, or negative effects. Although it appears that SGLT2i reduces the prevalence of atrial fibrillation and SCA, their effect on VA is not conclusive.

## 1. Introduction

The association between type 2 diabetes mellitus (T2DM) and an increased risk for cardiovascular morbidity and mortality is well-known [1,2]. T2DM particularly augments, by four to five times, the risk of developing heart failure (HF), one of the leading causes being the so called non-ischemic dilated cardiomyopathy (NIDCM), a form of cardiomyopathy associated with T2DM and characterized by important structural and functional changes in the myocardium, not determined by coronary artery disease and/or systemic hypertension [3,4,5]. Several pathophysiological pathways, related to the increased β-oxidation due to hyperglycemia, consecutive lipotoxicity (free fatty acid damage) of the myocardium, oxidative stress, activation of the sympathetic nervous system, and renin-angiotensin-aldosterone system (RAAS) with an increase release of angiotensin 2, endothelial dysfunction and altered calcium homeostasis are responsible for the occurrence of structural alterations in the myocardial tissue determining the transformation of the natural collagen matrix into a stiffer network, followed by enhanced apoptosis and fibrosis [6,7] which may favor the occurrence of malignant arrhythmias. Subsequently, the remodeling of the cardiac tissue occurs, leading to hypertrophy and enlargement of both ventricles, with elevation of preload and afterload and development of diastolic and systolic dysfunction [4].

Beside progression of HF, the principal causes for the increased mortality in NIDCM are ventricular arrhythmias (VA) and sudden cardiac arrest (SCA) [8,9]. Some of the responsible mechanisms are related to the alterations in the cardiac voltage-gated ion channels, namely the K^+^, Na^+^, and Ca^2+^ channels determined by T2DM, resulting in perturbations of action potential with insufficient repolarization followed by the occurrence of early and late after depolarization, thus increasing the risk of VA [10,11]. Other contributing factors that can trigger arrhythmias in NIDCM are hypoglycemia, hyperglycemia, acido-basic disturbances, and hypokalemia, which may also cause electrical and structural remodeling [10,12,13].

In the last decades, efforts have been made to develop new glucose-lowering agents (GLA) that not only offer a good control of blood glucose values (BGV) over a 24-h interval, but also have a favorable impact on cardiovascular (CV) morbidity and mortality, as well as protecting renal function [14,15,16]. Numerous scientific articles, reviews, and meta-analyses of the existing medical literature, as well as the results of clinical trials, have been published in the last decade. Most of them debate the beneficial cardiac effects of sodium-glucose cotransporter-2 inhibitors (SGLT2i), but also of other classes of medication such as thiazolidinedione, metformin versus sulfonylureas, glucagon-like peptide-1 receptor agonists (GLP1a), and dipeptidyl-peptidase 4 inhibitors (DPP4i) [3,16,17,18,19]. While there is an agreement that drugs for the last classes have little if any effect on VA or/and SCA, excepting perhaps metformin, which also lowers inflammation and oxidative stress, there are controversial opinions on SGLT2i agents [3,15,20,21]. This class of drugs is considered to have direct beneficial effects on the myocardium by improving the cardiac energetic metabolism, reducing oxidative stress, inflammation, fibrosis, and adverse ventricular remodeling, but also impacting cardiac ion channels and mediators that modulate cardiac electrophysiology, effects that could favor an antiarrhythmic effect, additionally ameliorating the renal function. While in the medical literature there is a consensus on the effectiveness of SGLT2i on CV morbidity, mortality, and hospitalization duration for HF regardless of the presence or absence of T2DM, the effects on VA and SCA are still a matter of dispute [15,16,22,23]. Considering that rigorous randomized clinical trials specifically on the effects of various GLA on malignant arrhythmias are missing, the available data mostly represent incidental observations, and that actual information relays mainly on observational studies (many of them retrospective) conducted on diabetic populations inhomogeneous from the point of view of ethnicity, age, risk factors and associated pathology, and the severity and extent of diabetes, the conclusions of these studies are heterogeneous and biased, although it seems that SGLT2i may exert a protective effect for SCA [16].

As more recent information could have become available with more recently published scientific works, the aim of our literature review was to analyze literature data collected from recent original articles (published in the last five years) regarding the effects of glucose-lowering agents on the risk of VA and SCA/SCD in patients with T2DM treated with various oral GLA, and to compare them with information already available in existing reviews and meta-analyses.

## 2. Materials and Methods

To identify suitable articles for our review, we searched in the international medical literature for scientific articles, published in English in the last five years on Pub-Med, Clarivate, and Google Scholar, on the topic of the effect of oral antidiabetic medication on SCA and VA in patients with T2DM. We used predetermined filters to select only articles conducted between 1 January 2018 and 31 December 2022, and published at least online, until 31 May 2023. We searched each data base individually by using the key words “type 2 diabetes mellitus”, “non-ischemic dilated cardiomyopathy”, “ventricular arrhythmias”, “sudden cardiac arrest/death”, “glucose lowering agents”, and “antidiabetic medication”.

Inclusion criteria: We selected only original articles published as full text in English that were available free of charge, referring to human adult population, and with a study group of over 1000 individuals.

Exclusion criteria were original articles having a study population of under 18 years old; referring to diabetes mellitus type 1; editorials, position papers, reviews, case reports, case series studies, published abstracts, poster and oral presentations, and author reply articles.

**Figure 1:**
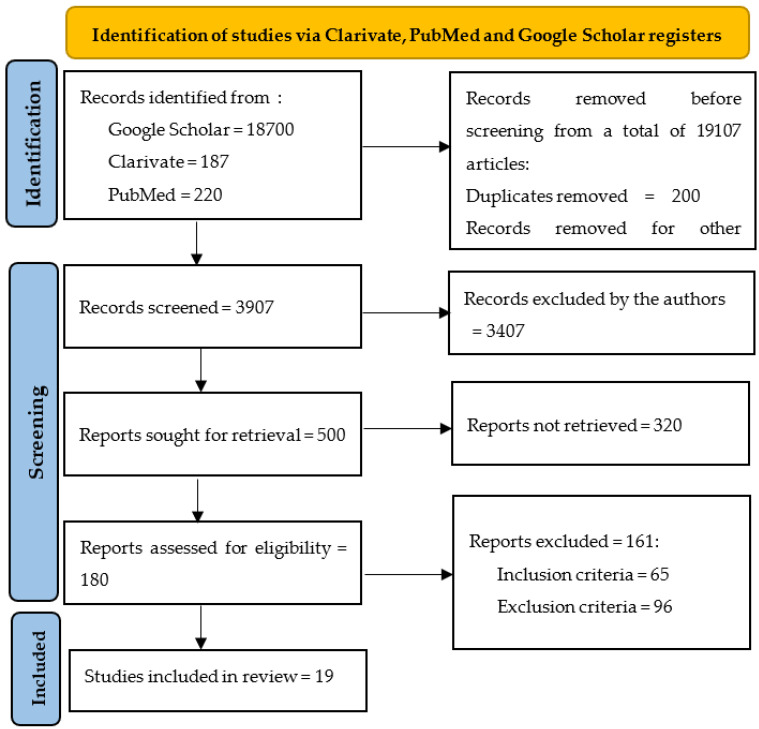
PRISMA chart reflecting the selection process.

Because several significant meta-analyses debating over the results of important clinical trials were published in the last few years, we used them only in the introduction or discussion section, but not in the proper analysis. All articles were introduced into Zotero and duplicates were eliminated. Initially, we identified 19,107 articles in the three researched databases, and we excluded 15,200 of them before screening (Figure 1). During the screening process, the remaining 3907 articles were individually assessed by all the authors, and 3407 records were excluded (not full-text available in English, conceptual or epidemiologic studies, not referring to humans, patients with type 1 diabetes mellitus, etc.). All full texts of the selected articles were reviewed to avoid the risk of biases (ROB). From the remaining 500 papers, a further 320 were excluded [24]. Using the inclusion/exclusion criteria set above, two authors (MT and CT) conducted a full assessment of the 180 identified scientific publications that remained, as depicted in a PRISMA flow chart (Figure 1). Any disagreement was resolved through discussion and with a third author (FV-M) if necessary.

This study was authorized by the Scientific Research Ethics Committee of our hospital (No. 206/7 September 2020).

## 3. Results

Of the nineteen original articles selected in our review, we identified seven debating the arrhythmogenic effects of second generation sulfonylurea compounds, and their properties were usually compared with metformin, both medication classes being commonly used for the treatment of patients with T2DM. We found only one study discussing the increased risk to develop VA/SCA exerted by thiazolidinediones, a group of drugs also frequently employed in the therapy of individuals suffering of T2DM. Referring to DPP4i, we identified two studies from 2022, both failing to demonstrate the effects of this class of medication on arrhythmias. Concerning GLP1a, although there were some studies relating to the beneficial effects on obesity, atherosclerosis, and CV complications, none of them debated the risk of VA or SCA. The most identified studies analyzed the influence of SGLT2i on CV outcomes and arrhythmias. All these studies are summarized in chronological order in Table 1.

### 3.1. Results Concerning the Arrhythmogenic Risk of Metformin Versus Sulfonylureas and Other oral GLA

Metformin is one of the most used GLA being recommended as first-line therapy by international guidelines. It is considered to reduce CV complications in T2DM patients, including the risk of developing supraventricular arrhythmias, although its effects on VA are not so well documented. Because, in most studies, metformin is studied in comparison with sulfonylureas, namely glimepiride, glyburide, and glipizide, other commonly used GLA, we decided to present them together. In prior studies, sulfonylureas were reported as being associated with increased CV morbidity and mortality, but not serious arrhythmogenic effects such as VA or SCA.

In their study, Weidner et al. [8] retrospectively analyzed the prevalence of VA, the re-hospitalization rate due to them, and the subsequent mortality by VA or/and SCA in patients with T2DM treated with metformin/sulfonilureea in comparison to non-diabetic individuals over a period of 2 years. They employed multivariable Cox regression models and propensity-score matching and concluded that T2DM is independently associated with an elevated all-cause mortality in patients with VA.

Another retrospective cohort study by Leonard et al. [25] analysed the safety of sulfonylureas, namely glyburide, glimepiride, or glipizide, concerning the risk of developing VA and SCA in patients with T2DM. Of all 519,272 sulfonylurea users, with a median age of 58.0 years, 632 developed VA/SCA/VA events (50.5% of them fatal) with a crude incidence rate of 3.6/1000 person-years. Compared with glipizide, VA/SCA risk hazard scores adjusted-ratios were 0.82 for glyburide and 1.10 for glimepiride. They concluded that glyburide may be associated with a lower risk for VA/SCA than glipizide.

A study by Dhopeshwarkar et al. [29] analyzed the risk of SCA and VA in patients with T2DM treated with second-generation sulfonylurea (glimepiride, glipizide, or glyburide) in two independent databases. They conducted two incident user cohort studies in two five-state Medicaid and Optum Clinformatics commercial claims and identified 624,406 and 491,940 sulfonylurea users, and 714 and 385 VA/SCA events in both databases. By employing Cox proportional hazards models, they failed to document statistically significant associations between VA/SCA and both glimepiride and glyburide versus glipizide. In this study, the incidence rates of VA/SCA in Medicaid (3.55 per 1000 p-y) and Optum (1.95 per 1000 p-y) populations were similar to those reported in the literature, being significantly higher than in the general populations, possibly explaining the two to fourfold increase in the risk for VA/SCA in patients with T2DM [29].

Starting from the premise that different oral GLA are associated with significantly different long-term risk of arrhythmias, other studies aimed to evidence the lower risk of arrhythmias in patients treated with metformin versus those treated with other GLA, especially with drugs from the second generation of sulfonylureas, but also with thiazolidinediones or DPP4 such as the observational study of Ostropolets et al. [31]. The authors used the IBM MarketScan Medicare Supplemental Database to explore the risk of VA, SCA, but also for atrial fibrillation in patients with T2DM treated with metformin versus other oral GLA and concluded that diabetic individuals being on metformin monotherapy had significantly lower risk of VA, SCA, bradycardia, but also atrial arrhythmias, in comparison to sulfonylureas as monotherapy. Mixtures between sulfonylureas and metformin were associated with a higher risk of arrhythmias in comparison with other combinations.

Another study on this topic is the cohort study of Lee et al. [33], who compared the risk of VA and/or SCA in patients with T2DM from Hong Kong, treated either with metformin or sulfonylurea at a 1:1 ratio. The matched cohort included patients older than 40 years without a history of myocardial infarction. Of them, 16,596 were metformin users and 16,596 sulfonylurea users. They were followed-up for almost 5 years and the authors concluded that sulfonylurea was associated with higher risk of VA or SCA than metformin with a hazard ratio of 1.90 [33].

A similar study from 2022 is the one published in 2023 by Islam et al. [38] conducted on adult patients newly treated with sulfonylurea monotherapy in comparison to those who received metformin monotherapy. From the UK’s Clinical Practice Research Datalink Aurum, the authors identified 92,638 new users of sulfonylurea and 506,882 new users of metformin. During the follow-up, among the sulfonylurea users a total of 279 VA/SCA events occurred (rate 25.5, per 10,000 person-years) versus 1537 VA/SCA events among metformin users (rate per 10,000 person-years = 18.5), evidencing an increased risk of VA/SCA in patients treated with sulfonylureas in comparison to metformin HR = 1.42.

A previous study [30], conducted by researchers from Taiwan, investigated the hypothesis that hypoglycemic medication induced episodes and also most of GLA themselves may increase the risk of VA/SCA in patients with T2DM. They analyzed data from 54,303 patients with T2DM, 1037 of them with documented episodes of hypoglycemia, and followed them for 3.3 ± 2.5 years. By employing a multivariate Cox hazards regression model, they evidenced a higher incidence of these events in the 29 subjects with VA/SCA in those with hypoglycemic episodes (adjusted HR: 2.42, *p* = 0.04).

### 3.2. Results Referring to Arrhytmogenic Risk of Thiazolidinediones

We only identified one study, by Leonard et al. [18], debating the arrhythmogenic effects of thiazolidinediones, a category of GLA with a lower cost used for the treatment of patients with T2DM, especially in low-income countries, approximately 300 million persons worldwide. The increased selectivity of these drugs for peroxisome proliferator-activated receptors in the myocardium may predispose the user to the occurrence of VA/SCA [18]. The authors performed populational cohort studies in five USA Medicaid programs and in Optum Clinformatics (a commercial health insurance database) in order to analyze the risk of developing VA or SCA in patients treated with rosiglitazone versus pioglitazone. By employing Cox proportional hazards regressions, the authors documented that the adjusted HR for VA/SCA among rosiglitazone versus pioglitazone users was 0.91 in Medicaid and 0.88 in Optum; thus, these two drugs appear to be associated with similar risks of VA/SCA.

### 3.3. Results Concerning Dipeptidyl Peptidase-4 Inhibitors

Starting from the hypothesis that arrhythmogenetic risk may be higher with less selective DPP4i, Dawwas et al. [34] separately researched the Medicaid Optum Clinformatics database in order to identify new users of saxagliptin, sitagliptin, and linagliptin (Optum only) and to estimate the risk of VA and SCA in these patients. By using Cox regression models, hazard ratios (HRs) were calculated, but the results were discordant. In Medicaid, the use of saxagliptin versus sitagliptin was associated with an elevated risk of VA/SCA (HR = 2.01), not sustained by the analysis of Optum database (HR = 0.79). No association between the use of linagliptin versus sitagliptin regarding the risk of VA/SCA was found (HR = 0.65) [34].

In opposition, another study performed by researchers from Taiwan analyzed the cardioprotective effects of DPP4i versus sulfonylureas in addition to metformin [32]. They conducted a nationwide cohort study and identified 37,317 patients with T2DM treated with DPP4i or SU in addition to metformin. These subjects were followed up for 2.1 years to evidence the increased risk of CV events, hospitalization, VA, SCA, and hypoglycemia. Compared with sulfonylurea users, patients treated with DPP4i showed a significantly lower risk of hospitalization for CV events—VA/SCA (HR 0.79). Sitagliptin and vildagliptin had a significantly lower risk of hospitalization for MACE, while saxagliptin had a borderline significantly higher risk of hospitalization for HF [32].

### 3.4. Results Concerning the Efficacy of SGLT2i for the Reduction of Arrhythmias Risk

A multinational observational study performed in 2018 [26] aimed to compare the SGLT2i, namely dapagliflozin, with DPP4i regarding the risk of major CV events, VA, SCA, CV mortality, hospitalization, and severe hypoglycemia during a year in patients with T2DM in a real-world setting. In nationwide registries in Denmark, Norway, and Sweden, the authors identified 40,908 patients with T2DM, 10,227 new users of dapagliflozin, and 30,681 of a DPP-4i. By employing Cox survival models, HR were estimated, and the authors concluded that dapagliflozin was associated with lower risks of CV events, arrhythmias, and all-cause mortality when compared to DPP4i in patients with T2DM.

Another study [27] conducted on 17,160 patients with T2DM followed for a median period of 4.2 years, of which 10,186 were without atherosclerotic CV disease, also analyzed the safety of dapagliflozin regarding major CV events, arrhythmias, and hospitalization rate. It was concluded that in patients with T2DM who were at risk for atherosclerotic CV disease, therapy with dapagliflozin did not impact that of major CV events when compared to placebo; however, it did determine a lower rate of CV death or hospitalization for heart failure [27].

A study from Taiwan [28] aimed to compare the risk for major CV events, including VA and/or SCA in 12,681 patients with T2DM newly treated with dapagliflozin versus empagliflozin by analyzing a multi-institutional electronic medical records database and performing multivariable Cox proportional hazard modeling. They failed to find statistically significant differences between these drugs.

Other researchers from Taiwan [9] studied the association between SGLT2i and new-onset arrhythmias and all-cause mortality and therapy with SGLT2i. They conducted a population-based cohort study by using Taiwan’s National Health Insurance Research Database, from which they identified a total number of 399,810 patients with newly diagnosed T2DM. Of them, 79,150 patients were newly treated with SGLT2i, and another 79,150 diabetic non-SGLT2i users served as controls. The SGLT2i group was associated with a lower risk of all-cause mortality (HR = 0.547) and arrhythmias (HR = 0.830; *p* = 0.0002).

Although SGLT2i have proven their efficiency in diabetic patients with heart failure, some physicians were concerned regarding a possible electrolyte imbalance due to osmotic diuresis, which could favor cardiac arrhythmias. Wu et al. compared 1056 diabetic patients on SGLT2i and 2119 without SGLT2i and found no statistical significant difference regarding VA and SCD [35].

By contrast, another single center study, which included 9609 diabetic patients followed-up for over 4 years, demonstrated that the treatment with SGLT2i reduced the risk of cardiac arrhythmias, VA, and the associated CV pathology [36].

A study that selected 152,591 diabetic patients on oral antidiabetic therapy from the UK Clinical Practice Research Datalink determined that SGLT2i, in comparison to other second- to third-line antidiabetic drugs, reduces SCA independent of sex, diabetes duration, or associated cardiovascular disease [37].

A more recent study, published in 2023, analyzed the incidence of heart failure, arrhythmias (SCA, VA, atrial fibrillation), and major CV events in patients with T2DM treated with SGLT2i [21]. The authors searched a federated electronic medical record database (TriNetX) for patients registered with T2DM during the previous 2 years. They identified 2,824,174 individuals, from which they selected two groups, each of 131,188 (SGLT2i users and non-SGLT2i users) and followed them up for 2 years. SGLT2i use was associated with a significantly lower risk of heart failure, all-cause mortality, incident atrial fibrillation, SCA, and composite of incident VA and SCA (HR = 0.76), and concluded that therapy with SGLT2i was associated with a significant reduction in the risk of incident HF, major CV events, and SCA but not of VA.

Starting from the hypothesis that some GLA may have beneficial direct effects on the myocardium and cardiac electrophysiology by impacting cardiac ion channels and their function, another study from 2023 [23], a nationwide Danish case-control study, investigated the effects of SGLT2i in comparison to GLP1a on cardiac arrests due to arrhythmias in patients diagnosed with T2DM between 2013 and 2019. Logistic regression models were employed and revealed a more reduced OR for cardiac arrests in SGLT2i treated patients when compared to GLP1a users, the result not being significantly influenced by gender, pre-existing cardiac disease, HF, and diabetes duration. The authors concluded that the use of SGLT2i in T2DM patients may reduce the risk of cardiac arrests due to arrhythmias in comparison with GLP1a [23].

## 4. Discussion

Patients with T2DM are exposed to a higher risk of developing HF, a condition associated with an increased risk of VA and SCA that may lead to an elevated CV mortality. GLA represents a heterogeneous medication, including several pharmacological classes, each of them with peculiar effects on CV system, either positive, neutral, and even unfavorable. According to the last five years of scientific studies and recent meta-analyzes, metformin remains among the most frequently recommended GLA. In addition to its well-established beneficial effect on HF, mortality, frequency, and duration of hospitalization, and protection of the renal function, SGLT2i have proven to have the best impact on atrial fibrillation and apparently on SCA, but without conclusive results on VA. Their protective effect seems to be less evident for these arrhythmias, but it is worth mentioning that there were no specific studies or clinical trials conducted to test these premises and therefore no definite conclusion can be drawn yet, further studies being necessary.

A peculiar interest on the CV effects of GLA has been granted since 2008, when the Food and Drug Administration (FDA), after the assumptions that rosiglitazone could increase the risk of developing myocardial infarction and HF, begun to require CV outcome trials as a condition to release licensing for new GLA. Numerous clinical trials have been performed and, subsequently, abundant medical literature has been written, especially in the years since the development of new classes of GLA with favorable CV effects. By contrary, less information on their potential association with VA and/or SCA are available [1,38,39,40]. However, there are several studies (19 of them analyzed in this review), and even some meta-analyses [15,16,20,41,42], most of them focusing on SGLT2, written in the last five years. Thus, the reported data are frequently contradictory.

The comprehensive reviews of Scheen and Savarese et al. [16,41], published in 2022, summarize extensive information on the interaction between the most important classes of GLA and VA, specifically SCA, as well as the current guidelines recommendations for the administration of each class [1,43,44].

Concerning the favorable CV effects of metformin, according to the extensive analyze of medical registries worldwide, the results of clinical trials, and large randomized cohort studies, metformin is associated with lower mortality when compared to controls (mainly sulfonylureas), and a more reduced risk of major CV events, thus being recommended by guidelines as a first-line therapy in patients with T2DM [1,16,41,42]. Accurate data on its effects on arrhythmias, especially on VA or SCA, are missing [16,42]. In our research, by analyzing seven studies who aimed to highlight if therapy with metformin decreases the risk of VA and/or SCA, most of them comparing diabetic patients treated with this drug to other therapies [8,25,29,31], although evidencing a beneficial effect on general mortality and rate of major CV events, and apparently on supraventricular arrhythmias, failed to emphasize a certain positive influence on VA and SCA [29,31,38]. However, most studies demonstrate that the incidence of VA, specifically SCA, is lower in patients treated with metformin in comparison to those receiving sulfonylurea products [31,33,38]. The studies analyzing the impact of second generation of sulfonylurea on VA and SCA, most of them realized in comparison to metformin, revealed a higher incidence of malignant arrhythmias, probably explained by the interference of this class of medication on ATP modulated K channels, altogether with potential episodes of hypoglycemia [31,33,38]. However, sulfonylurea is a heterogeneous group and differences regarding the arrhythmogenic risk between its drugs have been evidenced [18,25,29,31].

As for thiazolidinediones, we found scarce information in the medical literature, aspect also mentioned in well-known metaanalyses [16,41,42]. Though rosiglitazone was initially considered to trigger an increased CV risk by favouring the occurrence of myocardial infarction and HF, Leonard et al. evidenced a similar risk of VA and SCA for rosiglitazone and pioglitazone [25]. Thus, this class of GLA is not recommended in patients with increased CV risk, or evident cardiac disease [1,40,41,43].

While DPP4i have initially raised hopes regarding potential CV protection due to their pleiotropic effects, the results of large clinical trials evidenced even an augmentation of the HF prevalence [16,41]. Although the clinical trials evaluated various CV outcomes, there are few data concerning the risk of VA/SCA. We identified two studies from 2022 debating the potential increased risk of malignant arrhythmias or SCA [32,34], and both studies failed to document a significant increase or reduction of all-types of cardiac arrhythmias for none of the drugs belonging to this class (sitagliptin, saxagliptin, vildagliptin, lingliptin, alogliptin), but it is worth mentioning that the number of arrhythmic events was reduce.

Although GLP1a were also considered to exert pleiotropic effects on the CV system by reducing vascular inflammation and oxidative stress and by improving endothelial function and cardiac function [16,41,42], their effects on severe VA, specifically SCA, but also on new onset atrial fibrillation, HF, and the progression of renal disease, have not been sufficiently investigated and remain unclear [16,39]. Based on the current evidence, the European and American Guidelines recommend GLP1-Ra (or SGLT2i) as first-line treatment in patients with T2DM and high risk or established CV disease [1,43].

Over the last ten years, the results of multiple clinical trials have highlighted the beneficial effects of SGLT2i on the CV system, with an abundance of scientific articles debating this topic emerging in the medical literature. The pathophysiological mechanisms explaining their favorable CV effects are not completely understood. Based on this evidence, current European and American guidelines recommend SGLT2i as first-line therapy in patients with T2DM and increased risk or chronic CV disease [39,40,43]. According to expert consensus opinions and the updated version of HF guideline of the Heart Failure Association of the European Society of Cardiology, the actual evidence strongly recommends SGLT2i, namely dapagliflozin and empagliflozin, in patients with symptomatic HF with reduced ejection fraction (class I of recommendation, level of evidence A) [40]. Despite their impressive effects on HF outcomes in patients with T2DM and CV diseases, their antiarrhythmic effects have been less studied. Among the articles analyzed in this review, although a reduction of CV mortality, major CV events, aggravation and hospitalization for HF, and even of atrial fibrillation was observed in all of them, the majority failed to document a significant reduction of malignant arrhythmias or SCA. There were some studies that reported a decrease of VA and SCA in diabetic patients treated with SGLT2i, such the study of Chen et al. conducted on a large population from Taiwan [9], and another one conducted on a smaller population also from Taiwan, but followed for a longer time period [36]. Since there is no doubt that SGLT2i evidently reduces HF, and that cardiac arrhythmias are common in the HF population, the reduction of rhythm disturbances observed in this population could be mostly a consequence of intra-cardiac metabolic improvements leading to the augmentation of cardiac performance.

Study limitation: an important limitation is that we employed only three databases and only open access published original articles for this review. Because we aimed to elaborate a literature review, we did not perform statistical processing of the available data. Another limitation is that we did not analyze the effects of insulin on arrhythmias because there were controversies regarding the potential implication of hypoglycemia.

## 5. Conclusions

By analyzing the medical literature written in the last five years we noticed that data on the effects of GLA on malignant arrhythmias are less numerous, sometimes offering conflicting information and more difficult to interpret compared to those on supraventricular arrhythmias, especially of atrial fibrillation. Another aspect debated in the medical literature are the heterogeneous effects of various pharmacological class of GLA which may exert neutral, positive, or negative influences on malignant cardiac arrhythmias, these results being frequently influenced by the analyzed population/data-base and, the number of VA/SCD considered in different studies. However, best results were observed in some studies for SGLT2i, it has been assumed that they reduce the prevalence of atrial fibrillation and of SCA, but further studies and clinical trials are required to certify the potential favorable impact of this medication on malignant cardiac arrhythmias, namely VA and SCA.

## Figures and Tables

**Table 1:** Summary of studies analyzed in the review in chronological order.

Nr.	Article	Publi- cation Year	Study Population	Number of Patients	Objective of Follow Up	Observation
1.	Weidner et al. Observational study [8]	2018	Patients with T2DM, treated mostly with metformin and sulfonylureea	2411 patients with VA followed for 2 years	VA, SCA, CV events, all-cause mortality	T2DM represents an increased risk for VA, SCA and all-cause mortality.
2.	Leonard et al. Nonexperimental comparable safty study [25]	2018	USA, 30–75 years, new users of second generation sulfonylureea	519,272 adults	VA, SCA	Glyburide with 18% reduction of VA/SCA compared to glypizide.
3.	Persson et al. A multinational observational study [26]	2018	Adults with T2DM, new users either of dapagliflozin or of DPP4i	40,908 patients: 10,227 on dapagliflozin and 30,681 on a DPP4i	Major CV events, VA, SCA	SGLT2 were associated with lower CV morbidity and mortality, but not of arrhythmias
4.	Wiviott et al. A multinational observational study [27]	2019	Patients with T2DM, treated with dapagliflozin	17,160 patients followed for 4.2 years	Major CV events, VA, SCA	No reduction of major CV events, including VA and SCA, but a decrease on CV mortality and hospitalization
5.	Shao et al. A multi-institutional cohort study [28]	2019	T2DM patients, new SGLT2i users: 10,442 on dapagliflozin and 12,096 on empagliflozin	12,681 patients, mean age = 58.9 years,	Major CV events, VA, SCA	No significant difference between dapagliflozin and empagliflozin regarding major CV events
6.	Leonard et al. Cohort study [18]	2020	Patients with T2DM, from 5 USA states, new users of thiazolidinediones	500,091, aged 30–75 years, treated with thiazolidinediones	VA, SCA, precipitating hospital presentations	Rosiglitazone and pioglitazone are associated with a similar risk of VA and SCA
7.	Dhopeshwarkar et al. Two cohort studies from five US states [29]	2020	Adult patients with T2DM newly tretead with second generation sulfonylureea drugs	624,406 in Medicaid and 491940 in Optum	VA, SCA, precipitating hospital presentations	Conflicting results regarding VA/SCA for glimepiride and glyburide versus glipizide
8.	Chen et al., Populational based longitudinal cohort study [9]	2020	Patients newely diagnosed T2DM	399,810 P: 79,150 P with SGLT2i and 79,150 P without SGLT2i	SCA, VA	All cause mortality and arrhythmias were reduced.
9.	Hsieh et al., A nationwide cohort study [30]	2020	Adults, newly diagnosed T2DM, identified from the Taiwan National Health Insurance Database, treated with GLA among which sulfonylureea	1037 P with hypoglicemia due to GLA among wich sulfonylureea versus 4148 without hypoglycemia	VA, SCA	Hypoglicemia was assoviated with increased risk of VA/SCA
10.	Ostropolets et al. Observational study [31]	2021	Patients with T2DM, but without advanced disease/complications, treated with oral GLA, excluding insuline	645,785 P followed for 1 year	VA/SCA, atrial fibrillation	Patients on metformine monotherapy have a significanly reduced risk (34%) for VA compared to sulfonylureea.
11.	Wang et al. National cohort study [32]	2022	Adult pacients with T2DM from Taiwan, without advanced disease/complications	37,317 matched pairs of DPP4i and SU users, mean follow-up of 2.1 years.	Major CV events, VA, SCA and hospitalization	Sitagliptin and vildagliptin had a lower risk of hospitalization, VA and SCA
12.	Lee et al. Cohort study from Hong Kong [33]	2022	Pacients over 40 years old, without MI, treated either with metformin or sulfonylureeas	33,192 patients, followed 5 years	VA, SCA, death due to arrhytmias	Sulfonylureea was associated with higher risk of VA and SCA than metformin.
13.	Daawas et al. Population based cohort study [34]	2022	USA, subjects with T2DM, 30–75 years, followed over 1 year	48,388 patient treated with DPP-4i: saxagliptin, sitagliptin, linagliptin	VA, SCA	Discordand results regarding the association betweeeen SCA/VA and saxaglyptine compaired with sitaglyptine.
14.	Wu et al. Cohort study [35]	2022	Over 18 years, without cardiovascular pathology	1056 with SGLT2i inhibitors vs. 2119 controls	VA	No difference regarding VA between patients treated with SGLT2 inhibitors and those without SGLT2 inhibitors
15.	Jhuo et al. Cohort study [36]	2022	Followed minumum 4 years	9609–3203 with SGLT2i vs 6406 without SGLT2i	VA	Significant lower incidence of total cardiac arrhythmias and cardiovascular events.
17.	Eroglu et al. Cohort study [37]	2022	Newly treated diabetic patients. Median follow up was 2.6 years.	152591–15125 on SGLT2i treatment	VA, SCA	SGLT2i reduced all cause mortality but not SCD.
16.	Fawzy et al., Retrospective cohort study [21]	2023	Worldwide, all adult patients registered with T2DM during 1 January 2018 and 31 December 2019	131,189 patients treated with SGLT2i versus 131,189 without SGLT2i followed for 2 years	VA, SCA, atrial fibrillation, major CV events	SGLT2i reduce significantly CV events, SCD but not VA
18.	Júlíusdóttir et al. Nation wide case control study [23]	2023	Danish population	21708P with T2 DM—3618 with SCA—593 on SGLT2i	SCA	SGLT2i had lower risk for SCA in comparison to glucagon-like-peptide-1 receptor agonist.
19.	Islam et al. Cohort study from UK [38]	2023	Adult patients with T2DM, newly treated either with sulfonyl-urea or metformine	92,638 new user of sulfonylurea and 506,882 new users of metformine	VA	Sulfonyluree is associated with increased risk of VA

Legend: T2DM- type 2 diabetes mellitus, VA—ventricular arrhythmias, SCA—sudden cardiac arrest, CV—cardiovascular, SGLT2i—sodium-glucose cotransporter-2 inhibitors, DPP-4i—dipeptidyl-peptidase 4 inhibitors, SCD—sudden cardiac death.

## Data Availability

Not applicable.
